# Avian Expression Patterns and Genomic Mapping Implicate Leptin in Digestion and TNF in Immunity, Suggesting That Their Interacting Adipokine Role Has Been Acquired Only in Mammals

**DOI:** 10.3390/ijms20184489

**Published:** 2019-09-11

**Authors:** Eyal Seroussi, Martin Knytl, Frédérique Pitel, Daniel Elleder, Vladimir Krylov, Sophie Leroux, Mireille Morisson, Sara Yosefi, Shoval Miyara, Saibaba Ganesan, Mark Ruzal, Leif Andersson, Miriam Friedman-Einat

**Affiliations:** 1 Department of Animal Science, Agricultural Research Organization, Volcani Center, P.O. Box 15159, Rishon LeTsiyon 7528809, Israel; eyal.seroussi@mail.huji.ac.il (E.S.); yosefis@volcani.agri.gov.il (S.Y.); shoval.miyara@weizmann.ac.il (S.M.); saibabaabt@gmail.com (S.G.); markr@volcani.agri.gov.il (M.R.); 2 Department of Developmental Biology, Faculty of Science, Charles University, 12843 Prague, Czech Republic; martin.knytl@natur.cuni.cz (M.K.); vladimir.krylov@natur.cuni.cz (V.K.); 3 Department of Biology, Faculty of Science, McMaster University, Hamilton, ON L8S4K1, Canada; 4 GenPhySE, Université de Toulouse, INRA, ENVT, 31326 Castanet Tolosan, France; frederique.pitel@inra.fr (F.P.); sophie.leroux@inra.fr (S.L.); mireille.morisson@inra.fr (M.M.); 5 Institute of Molecular Genetics of the Czech Academy of Sciences, 14220 Prague, Czech Republic; daniel.elleder@img.cas.cz; 6 Department of Medical Biochemistry and Microbiology, Uppsala University, SE-75123 Uppsala, Sweden; Leif.andersson@imbim.uu.se; 7 Department of Animal Breeding and Genetics, Swedish University of Agricultural Sciences, SE-75007 Uppsala, Sweden; 8 Department of Veterinary Integrative Biosciences, College of Veterinary Medicine and Biomedical Sciences, Texas A&M University, College Station, TX 77843-4458, USA

**Keywords:** radiation-hybrid mapping, FISH-TSA, chicken, TNF, immune system, leptin, digestive tract, duodenum

## Abstract

In mammals, leptin and tumor-necrosis factor (TNF) are prominent interacting adipokines mediating appetite control and insulin sensitivity. While TNF pleiotropically functions in immune defense and cell survival, leptin is largely confined to signaling energy stores in adipocytes. Knowledge about the function of avian leptin and TNF is limited and they are absent or lowly expressed in adipose, respectively. Employing radiation-hybrid mapping and FISH-TSA, we mapped *TNF* and its syntenic genes to chicken chromosome 16 within the major histocompatibility complex (MHC) region. This mapping position suggests that avian TNF has a role in regulating immune response. To test its possible interaction with leptin within the immune system and beyond, we compared the transcription patterns of *TNF*, leptin and their cognate receptors obtained by meta-analysis of GenBank RNA-seq data. While expression of leptin and its receptor (*LEPR*) were detected in the brain and digestive tract, *TNF* and its receptor mRNAs were primarily found in viral-infected and LPS-treated leukocytes. We confirmed leptin expression in the duodenum by immunohistochemistry staining. Altogether, we suggest that whereas leptin and TNF interact as adipokines in mammals, in birds, they have distinct roles. Thus, the interaction between leptin and TNF may be unique to mammals.

## 1. Introduction

In mammals, leptin is a key adipokine that works in concert with other adipokines such as TNF (also known as TNFα) to regulate energy homeostasis [1,2]. TNF has a critical role in obesity-induced insulin resistance [3], in addition to its other functions [4]. The amount of leptin produced by the adipose tissue signals the amount of fat stores to the hypothalamus and peripheral tissues [1,2]. With respect to the immune response, various studies have shown that leptin modulates innate and adaptive immunity [5]. Leptin stimulates neutrophil chemotaxis and promotes macrophage phagocytosis and production of pro-inflammatory cytokines including TNF [6,7]. It is thought that leptin’s primary role in the immune system is adjusting the intensity of the immune response to the availability of energy stores [6]. TNF has a broader function in the immune system, which includes immune cell development and functional regulation. This is in addition to TNF’s general role in the control of survival, proliferation, differentiation and death of cells [8]. Taken together, leptin and TNF work in concert and stimulate each other’s expression both in the adipose tissue and in immune cells [9,10].

The identification of both TNF and leptin in birds has been hampered for decades, despite intensive efforts to find them [11,12] and they were suggested to be missing prior to recent discoveries [13,14]. For both *TNF* and leptin, erroneous sequences/annotations submitted to GenBank (accession nos. HQ739087 and AF012727, respectively) led to erroneous publications. This was much more pronounced for leptin (as summarized by Seroussi et al., [15]), but also true for *TNF* as denoted by Elleder and Kaspers [16], also resulting in misleading publications (see for example Rozenboim et al. [17]).

The motivation to keep searching for avian TNF and leptin was encouraged by the finding of their cognate receptors, including the chicken orthologs of the two mammalian *TNF* receptor genes (*TNFR1* and *2* [18,19], respectively) and the chicken leptin receptor (*LEPR* also known as *OBR*; [20,21,22]). Both the TNF and leptin receptors belong to the family of the cytokine type I receptors and were found to share functional motifs and consensus sequences with their mammalian orthologs. These include their corresponding ligand-binding domains, death domains of TNFR1 [23], and other consensus sequences implicated in their respective signal transduction pathways [18,19,20,21].

TNF and leptin were recently identified in several avian species including chickens [13,24,25,26,27,28]. In retrospect, it appears that the difficulty to identify these genes was due to the following characteristics: extreme GC-content (~70%), their location in genomic regions with low complexity repetitive and palindromic sequence elements, relatively low sequence conservation and low levels of expression. So far, characterization of the avian TNF and leptin has surprisingly revealed a very low or absent mRNA expression in adipose tissue as characterized under a variety of physiological conditions related to obesity, body growth, reproduction efficiency and feeding regimen [29].

Two similar versions of genuine chicken *TNF* and leptin genes were published. The first publication of both genes [13,15] lacked the 5′ regions, which were identified soon after [14,28]. For the *TNF*, an additional difference between the two versions is the length of a glycine stretch close the C’-terminal end, which is hard to resolve due to the low coverage with RNA-seq data from this GC-rich part of the gene. These difficult regions were characterized by optimized PCR amplification methods and Sanger sequencing [16]. The 5′ extended sequence of the original *TNF* is of high importance since it includes its predicted transmembrane domain of *TNF.* The 5′ extended sequence of leptin includes the expected signal peptide. However, this sequence is longer than that of orthologous leptins.

The human *TNF* gene maps to chromosome 6p21.3, contains four exons and spans about 3 Kilobases. The last exon shares similarity with lymphotoxin alpha (*LTA*, previously known also as *TNF-β*) [30]. These genes are linked to the major histocompatibility complex, class I, B (HLA-B) locus, analogous to their murine chromosomal position between the complement (class III) region and H-2D [31]. Except the ribosomal RNA genes cluster (also known as the nucleolus organizer region, NOR), which map near the centromere of chicken chromosome 16 (GGA16), nearly all protein coding genes presently mapped have been implicated in the immune responses [32,33]. These include the polymorphic major histocompatibility complex-B (MHC-B) and MHC-Y, both consisting of gene orthologs of the mammalian class I and II MHC genes [34,35]. In mammals, *TNF* maps within the MHC locus between lymphotoxin α and β genes. These three genes share some redundant activities, in addition to their unique functions [36]. Lymphotoxin α and β have not been found in chickens [11,12].

We have previously shown that the chicken *TNF* is expressed primarily in embryonic and adult spleens, as well as in monocytes and macrophages [13,14]. In these lymphocytes, *TNF* is induced by lipopolysaccharide (LPS) as a model for gram negative infections [14]. The avian leptin mRNA was primarily reported in brain tissues and, in some cases, also in gonads and adrenal regions [15,25,27,28], as well as a sporadic high expression in the liver [15]. Unlike the expression profile of chicken leptin, the chicken *TNF* profile of mRNA expression resembles that in mammals, except for low abundance in the adipose tissue [15]. Chromosomal mapping of *TNF* onto the chicken genome is crucial proof for its correct identification. This proof is especially important due to the difference between the avian and mammalian expression in the adipose tissue; the relatively low sequence similarity between chicken *TNF* and its mammalian and reptilian orthologs (~34% amino acid identity to human and ~39% amino acid identity to American alligator); and the erroneous annotation of chicken *TNF* sequence in GenBank. Thus, the primary aims of this work were to map and compare the chromosomal position of avian *TNF* with that of the mammalian *TNF* and to test if its mRNA expression profiling supports its possible interaction with leptin within the immune system and beyond.

## 2. Results

### 2.1. Mapping Chicken TNF onto Chicken GGA16 Using Radiation-Hybrid (RH) Panel

**Figure 1:**
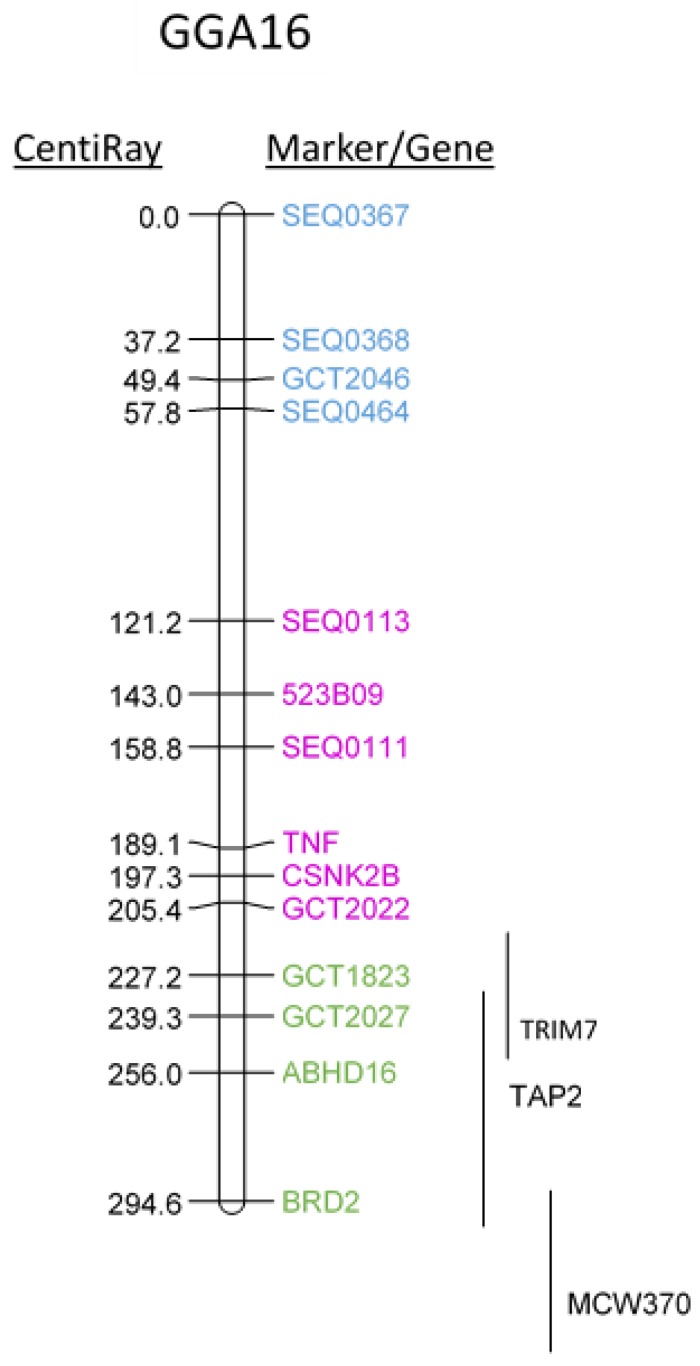
RH mapping of TNF to the q arm of GGA16. Positions of markers included in the comprehensive map are indicated with error bars on the right. Putative assignments [5,6] of nucleolus organizer regions (NOR; blue), major histocompatibility complex (MHC-Y; pink), and MHC-B (green) are indicated.

As a first indication for the location of chicken *TNF* within the chicken genome, we employed the well-established RH panel in Wg3hCl2 cells, prepared by fusing chicken embryonic diploid fibroblasts with hypoxanthine-guanine phosphoribosyl transferase (HPRT)-deficient hamster cells [37]. The analysis shown in Figure 1 located the chicken *TNF* close to *CSNK2B,* between SEQ0111 and GCT2022 markers of the MHC cluster Y on the q arm of microchromosome GGA16 [33]. This location was confirmed also by using PCR primers for *TRIM7.2*, *BRD2*, *TAP2* and *ABHD16A*, which are located within the MHC cluster on GGA16 in the current genome assembly (Table 1). The *CSNK2B* and *ABHD16A* genes are mapped onto a *TNF* genomic contig, also in crow (*Corvus cornix*) [14].

### 2.2. Mapping the Chicken TNF and its Syntenic Genes Using Fluorescent in Situ Hybridization with Tyramide Signal Amplification (FISH-TSA)

**Figure 2:**
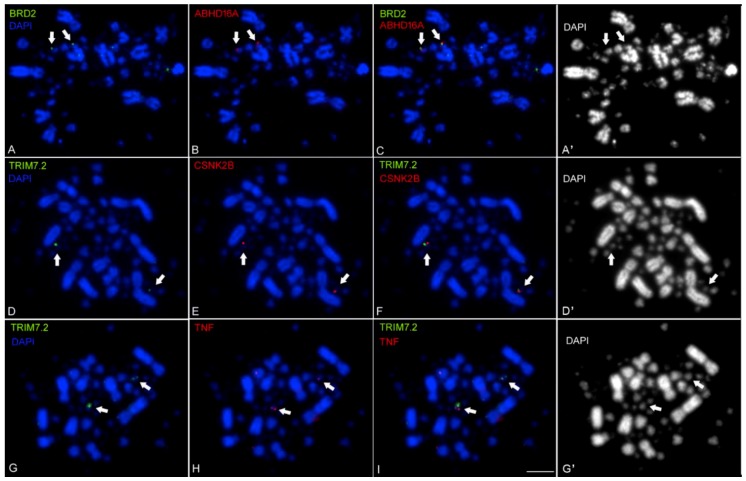
Detection of single-copy sequences in metaphase chromosomes by double color FISH-TSA. Somatic chromosomes prepared from a primary line of chicken embryo fibroblast (CEF) were used for hybridization with the indicated probes. Panels **C**,**F**,**I** were generated by merging of panels **A**,**B**,**D**,**E**,**G**,**H**, respectively, using red-green-blue color mode. For better visualization of the microchromosomes stained by 4’ 6-diamidino-2-phenylindole (DAPI), contrast-enhanced monochromatic images of **A**,**D**,**G** are shown in **A’**,**D’**,**G’**, respectively. Arrows indicate the two genes that were analyzed in each panel. Each gene locus was indicated in two copies on homologous chromosome pairs. Scale bar represents 10 µm.

To further confirm the localization of *TNF* on chicken GGA16, we used FISH-TSA on metaphase spreads of mitotic chromosomes. cDNA probes were prepared for *TNF* and for *BRD2, TRIM7.2, CSNK2B* and *ABHD16A* genes. Using dual-color labeling technique, the analysis showed that the three chicken genes, *TNF*, *ABHD16A* and *CSNK2B* were co-localized with either *BRD2* or *TRIM7.2* on a single microchromosome (Figure 2), thus, confirming the result of the RH mapping.

This mapping characterization summarized in Table 1 suggested the presence in chickens of an inflammatory region (MHC class III, between MHC class I and II), which is a homologous region between teleost fish and mammals [38]. In chicken, it is represented by the complement gene (*C4*). *C4* is closely associated with *TRIM 7.2*, *BRD2* and *TAP2* in the current GGA16 assembly.

### 2.3. Expression Profiling of mRNAs of TNF, Leptin and Their Cognate Receptors in Immune Cells

**Figure 3:**
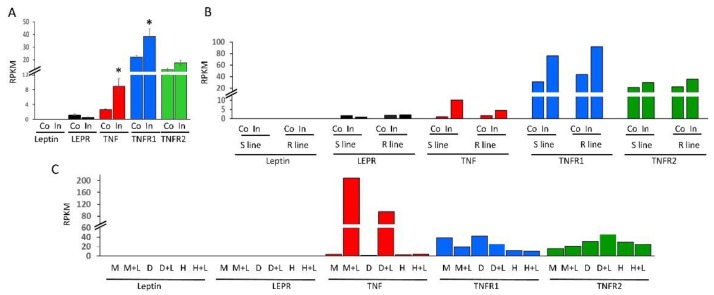
Meta-analysis of mRNA expression of chicken leptin, *TNF* and their cognate receptors in RNA-seq datasets from: **A**. * *p* < 0.05. Spleens of chickens intra-nasally administrated with PBS (Co) or very virulent infectious bursal disease virus (In); BioProject accession no. PRJEB7219 (*n* = 3 for each treatment). **B**. Spleens from 10 days old Leghorn lines susceptible (S line) or resistant (R line) to infection by Marek’s disease virus, five days after challenge with highly virulent Marek’s disease virus (In), or not challenged (Co) ([43]; BioProject accession no. PRJNA344896). **C**. Bone marrow-derived macrophages (M) and dendritic cells (D) from six-week-old broiler chickens and heterophils (H) isolated from blood of day-old broiler chicks (Ross), at the absence (M, D, H) or presence of lipopolysaccharide (+L) ([45]; BioProject accession no. PRJEB7475).

In mammals, *TNF* and leptin interact with each other to control immune functions and endocrine activities of the adipose tissue [39,40,41,42]. To test if a similar interaction exists also in chicken, we compared the mRNA expression patterns of leptin, *TNF* and their cognate receptors in tissues and immune cell lines by meta-analyzing the available RNA-seq data in the Sequence Read Archive (SRA) in NCBI. Sequence reads were counted using the SRA BLAST tool and sequences of chicken leptin, *LEPR*, *TNF*, *TNFR1* and *TNFR2* as baits (Figure 3).

Analysis of RNA-seq dataset from spleens of chickens four days after intranasal infection with bursal disease virus (IBDV) showed a significant induction of *TNF* and *TNFR1* (*p* ≤ 0.05); no induction of *TNFR2*; and no or low expression of leptin and *LEPR* (<1 RPKM), respectively (Figure 3A). Another analysis of spleens from white-leghorn strains is shown in Figure 3B. These strains were bidirectionally selected for high susceptibly and resistance to infection by Marek’s disease virus (MDV) [43,44]. Spleens of both the resistant and sensitive lines did not express leptin mRNA and lowly expressed *LEPR* mRNA, regardless of infection by MDV, while mRNAs of *TNF* and *TNFR1* were induced in the spleens of both sensitive and resistant lines (*TNF,* 10- and 3-fold; *TNFR1*, 2- and 1.5-fold, respectively). Since the data was obtained from pools of RNA samples from three birds of each treated group [43], statistical significance was not estimated. Nevertheless, analyzing this dataset suggested that leptin and *LEPR* are neither important for immune response nor to the degree of sensitivity of chicken spleens to viral infection. On the other hand, these results supported a role for *TNF* and *TNFR1* mRNAs in the infected spleens. Yet, an additional BLASTN search was performed on a dataset from primary cell lines of macrophages and dendritic cells derived from bone marrow and from heterophil cells isolated from blood (Figure 3C). The cell cultures were challenged with LPS for 24 h. *TNF* mRNA was induced 59, 60 and 2 folds in the cultured macrophages, dendritic and heterophil cells, respectively. *TNFR1* and *TNFR2* were slightly affected at the mRNA levels. Whereas in the immune cell lines, like in spleens, leptin and *LEPR* were undetectable and lowly expressed (below 0.4 RPKM in all of the samples), respectively. For chicken *TNF*, this analysis confirmed previous indications suggesting *TNF* implication in the immune response [13,14]. For the chicken leptin, this search suggested that in contrast to the prominent role of mammalian leptins in regulation of both innate and adaptive immune responses [42], the chicken leptin does not modulate an immune response.

### 2.4. Expression Profiling of Leptin, TNF and Their Cognate Receptors in Variety of Chicken Tissues

**Figure 4:**
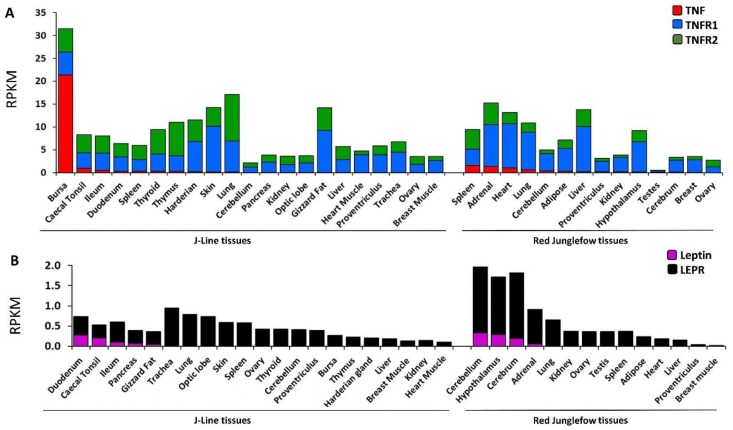
Meta-analysis of the expression patterns of *TNF*, leptin and their cognate receptors in adult J-Line and Red junglefowl chickens. A. Chicken *TNF*, *TNFR1* and *TNFR2* full-length sequences (accessions MF000729, NM_204439, and NM_001030779, respectively) were used as baits for BLASTN searching the SRA databases of 9 female 16 to 17 weeks of age J-line chickens (BioProject accession no. PRJEB12891) and two-year-old female and male red junglefowls (BioProject accession no. PRJNA204941). B. Chicken leptin and *LEPR* full-length sequences (accessions: LN794246 and NM_204323, respectively), were used as baits for BLASTN searching the same databases as in A. Results are presented as RPKM.

We extended our previous profiling of leptin, *TNF* and their cognate receptor expression patterns [13,15,28], using a meta-analysis of RNA-seq studies available in the SRA (Figure 4). Among the various tissues of adult J-Line and red junglefowl chickens, *TNF* was prominently expressed only in the bursa (above 20 RPKM), and it was also detected at a low level (<1 RPKM) in the spleen, adrenal, heart and lung. Similarly-low TNF mRNA levels were also observed in caecal tonsil, ileum and duodenum, which likely represent the high activity of these tissues in innate immune response through recruitment of immune cells [46,47]. Interestingly, the *TNF* receptors, *TNFR 1* and *2* seem to be ubiquitously expressed. This pattern confirmed the primary role of TNF in the immune system and suggested that, like in mammals, avian TNF affects survival of most cell types.

In addition to the known expression of the chicken leptin and *LEPR* in brain tissues of red junglefowl (cerebellum, hypothalamus and cerebrum; [15,28]), expression of these genes along the digestive system (duodenum, caecal tonsil, ileum, and pancreas) is reported in this study for the first time in chicken. This finding was based on querying the same datasets using the chicken leptin and *LEPR* sequences as baits. In J-line chickens, expression of leptin mRNA was observed in duodenum and caecal tonsil at rather low levels (0.28 and 0.20 RPKM, respectively) and at lower levels also in the ileum and pancreas (0.1 and 0.07 RPKM, respectively). This expression pattern suggests a role for chicken leptin in the digestive tract.

In mammals, expression of leptin in the stomach is well known. Therefore, our finding suggested that this site of leptin expression represents an important role.

### 2.5. Immunohistochemistry Analysis of Leptin in Chicken Duodenum

**Figure 5:**
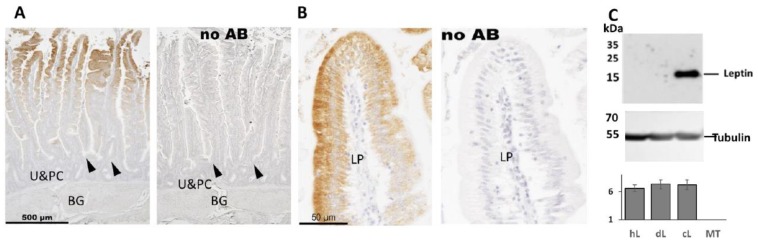
Immunohistochemistry analysis of leptin in chicken duodenum. **A**. Low power microphotographs of parallel non retrieved duodenum sections from mature leghorn chickens incubated with leptin antibody diluted from 1:1600 or processed without the primary antibody (no AB). Staining is observed in the mucosa in both enterocytes (absorbing cells) and goblet cells (mucosa secreting cells, distinctive by the presence of vacuoles). Crypts are indicated by arrow heads. U&PC are undifferentiated cells and Paneth cells. BG stands for Brunner’s gland. **B.** Higher magnification of a microvillus from B showing more clearly the absence of staining in the lamina propria (LP). **C.** The specificity of the leptin antibody was demonstrated by western analysis (top), using conditioned medium from 293 cells expressing exogenously administrated leptin minigenes of human, duck and chicken or mock transfected (hL, dL, and cL and MT, respectively) as described in details by Seroussi et al. [15]. Staining the same blot with tubulin antibody (middle panel) demonstrated that similar amounts of protein were loaded onto the gel wells, and the graph at the bottom indicated that a similar level of leptin activity was present in the three samples of conditioned medium (detected using LEPR based bioassay [15]).

The surprising finding of chicken leptin mRNA in tissues belonging to the digestive tissue indicates a possible role of leptin in chickens. To estimate if, like in mammals [48,49,50], chicken leptin is expressed in the gastric mucosa, we characterized duodenal leptin expression in mature female chickens by immunohistochemistry analysis (IHC) using chicken leptin-specific antibodies (Figure 5). Leptin was observed in the mucosa facing the lumen in both enterocytes and goblet cells fading gradually towards the crypts, where cells are less differentiated. Leptin was undetectable in cells below the crypts including Paneth cells, which represent the main epithelial cell type that secrete antimicrobial peptides and mucosal production of IgA. Paneth cells are the only cells in the small intestine of mammals normally expressing *TNF* mRNA [46]. The specificity of the antibody was demonstrated by western blotting (Figure 5C) applied with conditioned media (CM) of cells exogenously expressing human, duck and chicken leptins [15]. While leptin activity was similar in all three samples (bottom panel), only CM of chicken leptin-producing cells showed a signal of the expected size specific to leptin (top panel).

## 3. Discussion

We have mapped the chicken *TNF* gene together with additional four syntenic genes in mammals (*BRD2*, *TAP2*, *CSNK2B* and *ABHD16A*) to chicken chromosome 16q, within the MHC locus and demonstrated that these genes form a syntenic block also in chickens consistent with their co-localization in mammals and several other vertebrates [51]. This mapping position may define an MHC Class III inflammatory region in the chicken genome, which is a region of inflammation-related genes within the MHC cluster. The inflammatory region found to be similar in fish and mammals, but was represented in chicken and quail genomes only by the C4 gene [38].

The use of two different approaches for the mapping: FISH and RH-mapping strongly confirmed the genomic allocation of *TNF.* Interestingly, this position seems to be part of the GC-rich region observed within the GGA16 chromosome by a low DAPI staining [52]. Thus, the high GC-rich content (~70%) of the chicken *TNF* gene is likely to be shared by its neighboring genes and their intragenic sequences. Therefore, it is possible that the two closest neighboring genes of *TNF* in other vertebrates (lymphotoxin A and B), missing in chickens, would also be found; as happened in similar scenarios e.g., the leptin and *RBM28* genes in the centromeric GC-rich region of GGA1 [53].

Our meta-analysis of *TNF*, *TNFR1* and *TNFR2* mRNA expression in RNA-seq datasets in the SRA extended a previous study of *TNF* [13,14], indicating its implication in the immune response by showing that mRNAs of both *TNF* and *TNFR1* are induced in spleen and in immune cells upon virus infection and LPS treatment. The comparative mRNA expression analysis of *TNF* and its receptors shown here also suggested that TNF in the spleen and in immune cells has paracrine/autocrine mode of action in addition to its well established endocrine action. Unlike TNFR2, TNFR1 has a death domain, for executing TNF’s cytotoxicity and has high affinity to both the soluble and membrane bound TNF [54]. Our finding that *TNFR1* mRNA levels were more responsive to viral and LPS treatments than those of *TNFR2* is compatible with TNFR1 functions. Strikingly, in the same datasets, leptin mRNA was absent in the spleen and in immune cells under all of the experimental conditions. *LEPR* mRNA was lowly expressed and did not respond to treatments or to selective breeding toward resistance or sensitivity to infection. Since the role of the mammalian leptin is to communicate energy status to the immune system [40], this observation fits previous indications that chicken leptin is not a circulating signal of energy availability [15,27,28,29].

Profiling *TNFR1* and *2* mRNA expression in a variety of chicken tissues showed a wide range of expression of both receptors but with more abundant expression of *TNFR1* in most of the tissues. This characterization suggested a further role of chicken TNF not only in the immune system, but also in general cell survival, which is among its classical roles in mammals [4]. Since in mammals *TNFR2* is predominately expressed in cells of the immune system and endothelial cells [55], it is likely that in some of the chicken tissues low expression is contributed by endothelial and immune cells within the examined tissues.

The most surprising result in the current study was our finding of leptin and LEPR mRNA in the digestive system, opening a new frontier for chicken leptin research. In mammals, gastric leptin was discovered in 1998 by Bado et al. [48] and has been shown to be produced by the gastric chief cells in the stomach and by enterocytes and goblet cells in the duodenum [56]. The primary role of leptin in the digestive system is thought to be in gastric emptying and nutrient absorption activities [56,57]. In addition, gastric leptin also interacts with other gastric regulatory peptides such as ghrelin, cholecystokinin (CCK) and peptide transporter 1 (PepT1) [50] and affects CCK activation of vagus nerve afferents in the duodenum [58]. The possibility that gastric leptin serves as a short-term signal in the regulation of food intake, has also been suggested in mammals [57], complementing the long-term regulation of appetite by leptin secreted from the adipose tissue [2]. In mammals, expression of leptin in the stomach is well known. Therefore, our finding suggested that this site of leptin expression represents a role for leptin that is common among many species.

The immunostaining of leptin in the duodenum and the finding of its presence in mucosal cells (enterocytes and goblet cells), provided a critical proof that the low expression of leptin mRNA (0.3 RPKM) in J-line duodenum is significant. Our finding of leptin in the digestive system raised the possibility that chicken leptin may operate in short term regulation of appetite based on the digestive activity. This may take place either via the afferent vagus nerve or through the blood circulation or both. However, more work is needed to evaluate this possibility.

## 4. Materials and Methods

### 4.1. RH-Mapping

PCR amplifications were carried out for each marker (Table S1) in 15 μL solution containing 25 ng DNA from the RH panel [37], 0.4 μM of each primer, 0.25 units Taq polymerase (GoTaq, Promega Madison, WI, USA), 2 mM MgCl2, 0.2 mM dNTP, using the Applied Biosystems 2720 thermal cycler. The first 5 min denaturation step was followed by 35 cycles, of denaturation at 94 °C for 30 s, annealing at Tm (see Table S1) for 30 s and elongation at 72 °C for 30 s. Each marker was genotyped twice and a third genotyping was performed in case of discrepancy between the first two determinations. The RH map was built as previously described [59] using the Carthagene software [60] and drawn with MapChart 2.0 [61].

### 4.2. FISH-TSA Analysis

#### 4.2.1. Chromosome Preparation and Cell Culture

Primary line of chicken CEF cells, were prepared from 10-day old pooled chick embryos (males and females) of an inbred White Leghorn strain [62] as previously described [63]. Cells were maintained at 37 °C and 5% CO2 in a mixture of two parts Dulbecco’s modified Eagle’s medium and one part F-12 medium, supplemented with 8% fetal calf serum, 2% chicken serum, and antibiotic-antimycotic solution (Sigma-Aldrich, St. Louis, MO, USA). Chromosome metaphase spreads obtained from the CEF cell culture (passage 5) were prepared according to Courtet et al. [64]. Cell suspension was spread onto a clean glass microscopic-slide one day before use for FISH-TSA and stored overnight at −20 °C.

#### 4.2.2. cDNA Probe Preparation and Labelling

Total RNA was isolated from chicken DF-1 cells [65] using TRI reagent (Sigma-Aldrich) and converted to cDNA with the SMART RACE protocol (Clontech, Palo Alto, CA, USA). The probes for the chicken genes were prepared by PCR amplification from the cDNA using the primers listed in Table S2, (see also Sequence S1 and Figure S1 describing our genuine *ABHD16A* sequence) and conditions previously described [14]. The PCR products were separated by agarose electrophoresis and purified using the Qiaex II gel extraction kit (Qiagen, Valencia, CA, USA). The cDNA probes (1 µg each) were labeled by random primed method using the DecaLabel DNA Labeling Kit (Thermo Fisher Scientific, Waltham, MA, USA) according to manufacturer’s instructions.

#### 4.2.3. FISH-TSA

Double color FISH-TSA protocol was adapted from Krylov et al. [66] with minor modifications described in Knytl et al. [67]. ABHD16A, CSNK2B, and TNF probes were labeled by Digoxigenin-11-dUTP (Roche, Mannheim, Germany) and hybridized to chromosomal DNA overnight at 37 °C. Signals were detected by anti-digoxigenin antibody conjugated to horseradish peroxidase (Anti-Digoxigenin-POD, Fab fragments, Roche), and amplified by tetramethylrhodamine (TMR) with TSA Plus Fluorescein and TMR System Kit (PerkinElmer, Inc., Waltham, MA, USA). BRD2 and TRIM7.2 probes labeled by Biotin-16-dUTP (Roche) were detected by streptavidin-POD, conjugate (Roche), and signal was amplified by fluorescein (TSA Plus Fluorescein and TMR System Kit, PerkinElmer Inc., Waltham, MA, USA). Hybridization mixture containing both hapten-labeled probes was applied on chromosome slide as previously described [68]. Targeted single-copy gene fragments were visualized in sequential rounds of incubations as detailed before [69] in the following order: (1) streptavidin-POD, TSA fluorescein; (2) anti-digoxigenin-POD, TSA TMR. Between the sequential steps 1 and 2, slides were incubated in 1% hydrogen peroxide (H2O2/PBS) for 30 min at room temperature, then three washes in PBS for 5 min at room temperature, and a final wash in TNT buffer (0.1 M Tris-HCl, 0.15 M NaCl, 0.05% Tween-20, pH 7.5) for 5 min at room temperature. Chromosomes were counterstained by DAPI with antifade (Cytocell, Cambridge, UK).

#### 4.2.4. Microscopy and Processing of FISH-TSA Images

FISH-TSA images were captured with camera DFC 7000T (equipped with a black-and-white CCD-Chip (Leica, Wetzlar, Germany)) coupled to an epifluorescence microscope Leica DM6B equipped with a set of three narrowband fluorescent filters. Digital pseudocolored images (blue for DAPI, green for biotinylated probe, red for digoxigenated probe (described in Section 4.2.2), were merged in red-green-blue channel mode and processed by Adaptive Contrast Control Scientific Image Analyzer software (Sofo ACC 6.2, Brno, Czech Republic). and Adobe Photoshop (CS7), Adobe Systems, San Jose, CA, USA.

### 4.3. Bioinformatic Analysis

Deducing the sequence of the chicken ortholog of *AHBD16A* was performed using the human *AHBD16A* sequence for BLAST searches using RNA-seq datasets (study PRJEB7620). Sequence reads were assembled with CLC genomics workbench, Qiagen, Bustehrad, Czech republic and with Lasergene SeqMan (DNASTAR, Madison, WI, USA).

### 4.4. Animals and Tissue Sampling

Commercial females of the Leghorn breed (layers) were purchased from commercial husbandries (Hasolelim, Israel) at the age of 1 d and grown at the Volcani Center according to recommended husbandry and feeding conditions (NRC 1994) with free access to food and water. Maintenance conditions and feeding formulas were according to the Lohmann guideline (http://www.hylinena.com/userdocs/products/lohmann_brown_lite_commercials_2011.pdf), as detailed previously [29]. At the age of first egg, lay (about four months of age) tissue samples were snap-frozen in liquid nitrogen, after neck dislocation. All animal procedures were carried out in accordance with the National Institutes of Health Guidelines on the Care and Use of Animals and Protocol IL732/17 (1.11.17), obtained by the Animal Experimentation Ethics Committee of the Agricultural Research Organization, Volcani Center.

### 4.5. Antibodies and Western Analysis

A custom-made antibody directed against chicken leptin-peptide (amino acid sequence: PPRAEKLRADARSLSRTLSARLGD) was prepared in rabbits and affinity purified by HY Laboratories LTD, Rehovot Israel. Anti alpha tubulin antibody was purchased from ABCAM (ab89984, Zotal, Tel-Aviv, Israel).

Protein concentration was measured by Bradford assay (Sigma, Yavne, Israel). Total proteins (20 mg) were subjected to 12% SDS-PAGE and transferred to a nitrocellulose membrane.

### 4.6. IHC Analysis

Chicken duodenum was fixed in buffered formalin for ~48 h. Fixed specimens were dehydrated through graded ethanol concentrations, cleared in xylene and embedded into paraffin. Five micrometers longitudinal sections were prepared and mounted onto positively charged glass slides. For immunohistochemical staining sections were deparaffinized in xylene and rehydrated through descending ethanol concentrations. Rehydrated sections were incubated with rabbit polyclonal antibodies to chicken leptin diluted 1:600 in TBST (10mM Tris-HCl, 150 mM NaCl, 0.1% Tween-20, pH 7.5) for 1 h at room temperature. After washing in TBST (5 times for 2 min) sections were incubated with anti-rabbit ZytoChem Plus HRP-Polymer (Zytomed Biotest, Kfar Saba, Israel) for 30 min. Then sections were washed in TBST and peroxidase activity was revealed by incubation in a mixture of 0.05% diaminobenzidine hydrochloride (Sigma-Aldrich, Rehobot, Israel) with 0.03% hydrogen peroxide in 0.1 M Tris-HCl, 10 mM imidazole (pH 7.5). Sections were slightly stained with Gill’s hematoxylin, dehydrated, cleared in xylene and mounted in DPX (Sigma-Aldrich). Digital microphotographs were prepared using SPOT InSight CMOS camera attached to BX51 microscope (Olympus, Japan). This analysis was conducted by Smart Assays, Ness Ziona, Israel.

### 4.7. Statistical Analyses

Statistical analyses of the qRT-PCR analysis and leptin bioassay were performed by one-way ANOVA and Tukey-Kramer honestly significant difference test (*p ≤* 0.05); means ± SEs are reported.

## Figures and Tables

**Table 1:** Summary of mapping positions.

Gene Name		Position RH-Mapping (CentiRay)	Linkage by FISH	Position GGA16	Position HSA6
*TRIM 7.2*	*Tripartite motif containing 7*	200–240	CSNK2B, TNF	2,482,253..2,492,415	
*BRD2*	*Bromodomain containing 2*	294.6	ABHD16A	2,574,363..2,583,216	32968594..32981505
*TAP2*	*Transporter 2, ATP-binding cassette*	220–300		2,601,162..2,604,724	32821833..32838823
*C4A*	*Complement 4*			2,610,038..2,624,392	31982057..32002680
*TNF*	*Tumor Necrosis Factor*	189.1		Unknown	31575565..31578336
*CSNK2B*	*Casein kinase II subunit beta*	197.3		Unknown	31665880..31670070
*ABHD16A*	*Abhydrolase domain containing 16A*	256		Unknown	31686949..31703360

## Supplementary Materials

Supplementary materials can be found at https://www.mdpi.com/1422-0067/20/18/4489/s1.
