# Supplementary material for: Avian Expression Patterns and Genomic Mapping Implicate Leptin in Digestion and TNF in Immunity, Suggesting That Their Interacting Adipokine Role Has Been Acquired Only in Mammals
Source: Int J Mol Sci. 2019 Sep 11;20(18):4489. doi: 10.3390/ijms20184489 (PMC6770569; doi:10.3390/ijms20184489)
Supplement: Supplementary file 1 [file ijms-20-04489-s001.pdf]

Sequence 1: ABHD16A predicted transcript sequence

ATGGCGAAGCTGCTGAGCTGCGTTCTGGGCCCGCGCCTCTACCGCGTCCACCGGCGGCGCGCCGAGGGG
GGGGAGCGCCGCGAGCCGAGCTGGGACTCTTACTACCAACCGCGGGGCCTGGAGAAGCACACGGACAG
CGTCCTGGCTCTGGCCTCGGTGCTGTGGTCCATTTCATACTACGCTTCGCCTTTGGCCGTGTTTTATCTGTA
CCGGAAAGGGTTGCTGACCATGTCCCGCGTGGTGCCGCTGTCGCACTGCGCCGCAACCATCGCGCTGCT
GCTGGCGGGGGTGGCCTGTCTGAGAGGGCTAGGCCGCTGGAGCAACCCGCAGTACGTGGAGTTCATCAC
GGTGCTGGAGGACAGCCACCGACTGGGGACCCCCGAGACCAAGCGCAAGTTGGCCGCATACACCTTCG
ACTTCCGCAGCTGGCCCGTTGACTTCCGCTGGGACAGCGCCGGGCAGCCCTGGGGTGGGGCTAAAGCCG
TCCCATTGCTCCGTGCCCCCCCACCTCGGCACCACCGTGGGGTCCTGGCTGCCATCCGGAAGCTTCCGTG
CCAGCTGGCCAGCCTGTTGCTGGCGCACACCCTGGGCCGCCGGCTGCTGTTCCCGGGTTCGGTGCGGCTC
CTGCAGAGGGCGCTGCTGCCGGCGCTGCTGCACGGACAGGCGCGCCTCGTCGAGGAGTGGGGCGGGCA
GCGTGCGAAGCTGCAGGCGTGCGATGGGAACCACATCGATACGATGTTCGTGGACCGACGGCACCGCC
CCGACCCCCGCGGGCAGAAATTGGTGATCTGCTGTGAGGGCAACGCCGGCTTCTATGAGGTCGGATGTC
TCTCCACCCCCCTCGAGGCCGGGTACTCCGTGTTGGGTTGGAACCACCCCGGCTTTGCAGGCAGCACTGG
GGTTCCTCTGCCGCAGAGCGAGGCCAACGCGATGGACGTGGTGCTGCAATACGCCCTGCAGCGCCTCGG
CTTCGCCCCCAGGGACCTCTTCATCTACGCGTGGTCCATTGGGGGCTTCACCGCCACGTGGGCAGCCATG
TGGTACCCCGATGTGGGGGGGTTGGTTCTGGACGCCTCCTTTGACGACCTTCTGCCTTTGGCCCTCAAGGT
GCTGCCCCGCAGCTGGCGCGATTTGGTGACGCTGACGGTTCGCCAACACCTCAACCTCAACAACGCAGA
GCAGCTCTGCAGGTATCAGGGCCCGGTGCTGCTGGTGAGGAGGACGCGGGATGAGATCATCACCACCA
CGACCCCCGATGACATCGCCTCCAACCGCGGCAATGATTTACTGCTCAAACTGCTGCAGTTCCGGTACCC
CAAAATCATGGCGGAGGAGGGGCTGCGCGTGGTGAAGGAGTGGTTGGGGGCCGCCACCCCCAGCGAGG
AGGAGGCGGTGCTGCAGCGCTGCCCRTTGGACTCCGATTGGTGCCTCTCAGCGCTGGGGGGCTTCGCCG
GGGAGAGCCCCCCCCCTTCCCATGGGCTTTGGGGGAGGAAATGACGGCGGAGCAGCGCCGGATGTTG
GCGTTGTTTTTGGCTCAGAAGCACCTGCAGAACTTCGAGGCCTCGCACTGCACCCCTTTGCCCCCCCCCG
CCTTCCGCCTCCCATGGACTCTGTGA

Complete coding sequence of the chicken *ABHD16A* gene was deduced from Genbank available RNA-seq data in the SRA as described in the Material and Methods section.

```
chicken         MAKLLSCVLGPRLYRVHRRRAEG-------------GERREPSWDSYYQPRGLEKHTDSV    47
human           MAKLLSCVLGPRLYKIYRERDSERAPASVPETPTAVTAPHSSSWDTYYQPRALEKHADSI    60
alligator       MAKLLSCVLGPRLYRVYRERGPARLPRDGDEATEGSPASRESSWDSYYQPRSLEKHSDSI    60
                **************:::*.*                   :. ***:*****.****:**:

chicken         LALASVLWSISYYASPLAVFYLYRKGLLTMSRVVPLSHCAATIALLLAGVACLRGLGRWS    107
human           LALASVFWSISYYSSPFAFFYLYRKGYLSLSKVVPFSHYAGTLLLLLAGVACLRGIGRWT    120
alligator       LALASVLWSISYYTSPLAFFYLYRKGYLTMSKVVPFSHYAATMLFLLAGVACLRGIGRWT    120
                ******:******:**:*.******* *::*:***:** *.*: :**********:***:

chicken         NPQYVEFITVLEDSHRLGTPETKRKLAAYTFDFRSWPVDFRWDSAGQPW---GGA--KAV    162
human           NPQYRQFITILEATHRNQSSENKRQLANYNFDFRSWPVDFHWEEPSSRKESRGGPSRRGV    180
alligator       NPQYIQFITILEDSHRIGTPEIKKKLASYNFDFRSWPVDFRWDEVSSRSDS------RGI    174
                **** :***:** :**  : * *::** *.*********:*:. ..          :.:

chicken         PLLRAPPPR--HHRGVLAAIRKLPCQLASLLLAHTLGRRLLFPGSVRLLQRALLPALLHG    220
human           ALLRPEPLHRGTADTLLNRVKKLPCQITSYLVAHTLGRRMLYPGSVYLLQKALMPVLLQG    240
alligator       SLLQPPPKHRSTANGFLNAVKKLPCQIASYIVAHTFGRRMLYPGSVYLLQKALMPMLLQG    234
                 **:  * :       .*  ::*****::* ::***:***:*:**** ***:**:* **:*

chicken         QARLVEEWGGQRAKLQACDGNHIDTMFVDRRHRPDPRGQKLVICCEGNAGFYEVGCLSTP    280
human           QARLVEECNGRRAKLLACDGNEIDTMFVDRRGTAEPQGQKLVICCEGNAGFYEVGCVSTP    300
alligator       QARLVEEWGGKRAKLLACDGNEIDTMFMDRRGTSEPQGAKLVICCEGNAGFYEVGCLSTP    294
                ******* .*:**** *****.*****:***   :*:* *****************:***

chicken         LEAGYSVLGWNHPGFAGSTGVPLPQSEANAMDVVLQYALQRLGFAPRDLFIYAWSIGGFT    340
human           LEAGYSVLGWNHPGFAGSTGVPFPQNEANAMDVVVQFAIHRLGFQPQDIIIYAWSIGGFT    360
alligator       LDAGYSVLGWNHPGFAGSTGVPLPQAEANAMDAVVQFAIHRLGFQPDDIILYAWSIGGFT    354
                *:********************:** ******.*:*:*::**** * *:::*********

chicken         ATWAAMWYPDVGGLVLDASFDDLLPLALKVLPRSWRDLVTLTVRQHLNLNNAEQLCRYQG    400
human           ATWAAMSYPDVSAMILDASFDDLVPLALKVMPDSWRGLVTRTVRQHLNLNNAEQLCRYQG    420
alligator       ATWAAMTYPEISALVLDASFDDLVPLALKVMPESWRGLVTRTVRQHLNLNNGEQLCRYQG    414
                ****** **::..::********:******:* ***.*** **********.********

chicken         PVLLVRRTRDEIITTTTPDDIASNRGNDLLLKLLQFRYPKIMAEEGLRVVKEWLGAATPS    460
human           PVLLIRRTKDEIITTTVPEDIMSNRGNDLLLKLLQHRYPRVMAEEGLRVVRQWLEASSQL    480
alligator       PVLLVRRTKDEIITTTVPEDIMSNRGNDLLLKLLQHRYPKVMGEEGVRMVREWLEASSPM    474
                ****:***:*******.*:** ************.***::*.***:*:*::** *::

chicken         EEEAVLQRCPLDSDWCLSALGGFAGESPPPFPWALGEEMTAEQRRMLALFLAQKHLQNFE    520
human           EEASIYSRWEVEEDWCLSVLRSYQAEHGPDFPWSVGEDMSADGRRQLALFLARKHLHNFE    540
alligator       EEVAVCSRYEVEDDWCMSVLRSYRDQHGPDFPWSVGDDMMPEGRRQLALFLARKQLQNFE    534
                ** :: .*  ::.***:*.* .:  :  * ***::*::*  : ** ******:*:*:***

chicken         ASHCTPLPPPAFRLPWTL      538
human           ATHCTPLPAQNFQMPWHL      558
alligator       ATHCTPLPHHNFRLPWRL      552
                *:******   *::** *
```

Figure S1.Alignment of the predicted protein sequence of chicken ABHD16A (Figure 1S) with those of human (GenBank accession AQY76647) and alligator (GenBank accession XP_006037708), using Clustal Omega software. Periods, colons and asterisks indicate weakly similar, strongly similar and identical amino acids, respectively.

Supplementary Table 1, Primers used for RH-mapping

Marker	Forward	Reverse	TM (°C)
SEQ0367	AGAGGGAAGGAGCGAGAG	GCGACCAAAGGAACCATAAC	55
SEQ0368	CCAGTGCGGTAACGCAAG	GCGAGATTTACACCCTCTC	55
GCT2046	GCAATTATTCCCCATGAACG	AGTTCGACCGTCTTCTCGAC	52
SEQ0464	GGTGCCGAAAGTCAGACAAC	AAAGCGACCCTCAGACAGG	58
SEQ0113	TCTCTGCACCCAACCTATCC	TCAGAAGTGAGAGCGAAAGC	55
523B09	ACGTTCCCTTCTCACTCTGC	CATCTGACACCCTGCCTTC	50
SEQ0111	TACTTCCTGACCGGGATGAC	CTTCTGTTGTAGCGCACCTG	55
TNF	TCGTGGCATCGTCCTCTCAG	ACCAATTTGTTCCTTCTCAGCAC	58
CSNK2B	GGGAGGCGATGGTGAAGCTG	GCACGAATTGGTTTGCGGGGC	60
GCT2022	CGGAACTTCGCCTTCATCTC	AGTCGTTGTCGTGGTGCTG	58
GCT1823	CTGGTGATCCCGGAGAAG	ATGGCGAAGGCGATCTTG	60
GCT2027	ATTACATCGCGGTGAAGGAG	TGAGCAGATGGATGATCTCG	58
ABHD16	CCTGTTGCTGGCGCACAC	CGCTATGGGTCCTGACCTCCTC	55
BRD2	GAGCTTCGATTTCCTGTGC	GTGTGGGCTCTTCCAGTTC	60
MCW370	AAAGAGGAGAGTAGTTCACG	CCCACCTCATCATGCATTCC	55
TAP2	TACGAGCACCGCTACCTG	AGAGATGAAGCCCAAAGCAC	55
TRIM7	GTCCCTGTGTGAGAGACCCCG	AGCGAGGAGCCCACGTAGAAG	58

Supplementary Table 2, Primers used for FISH analysis

Gene name	Accession numbe/other reference	Forward	Reverse	Probe length (bp's)
TNF	MF000729	GTGGGCGGTGCGGCCATA	TTAATCCACTCCCACCACCCCG	697
CSNK2B	NM_001257204	GCTCCGAGGAGGTGTCGTGGATCT	TTCACGGGGCTCTTGAAGTTGCTG	628
ABHD16A	Suplementary sequence	GGCCTCAGTGCTGTGGTCCATT	GAGAGGCACCAATCGGAGTCCA	1284
BRD2	NM_001030674	TCAAGTCGCTGCACTCCACTG	AGGACGAGGAGCTGGAACTGG	1560
TRIM7.2	NM_001099354	GTGCGTTGTGTGCCACCTCT	AGCGAGGAGCCCACGTAGAAG	1068
